# Changes in thyroid function levels in female patients with first-episode bipolar disorder

**DOI:** 10.3389/fpsyt.2023.1185943

**Published:** 2023-11-13

**Authors:** Xiuhua Song, Yufang Feng, Lei Yi, Baoliang Zhong, Yi Li

**Affiliations:** ^1^Department of Psychiatry, Mental Health Center of Qingdao City, Qingdao, Shandong Province, China; ^2^Department of Psychiatry, Affiliated Wuhan Mental Health Center, Tongji Medical College of Huazhong University of Science and Technology, Wuhan, Hubei Province, China

**Keywords:** bipolar disorder, mania, depression, thyroxine, thyroid hormone

## Abstract

**Objectives:**

The identification of molecular biomarkers for bipolar disorder is anticipated to greatly improve the diagnosis and treatment of this disease. The objective of this case–control study is to determine whether the blood thyroid hormone levels in bipolar disorder patients are associated with different types of first onset.

**Methods:**

From August 1, 2020 to July 31, 2021 a total of 120 female patients diagnosed with bipolar disorder and hospitalized at Qingdao Mental Health Center were recruited as the case group, including 60 patients with depression as their first onset (depression first-episode group, DF) and 60 with mania/hypomania as their first onset (mania/hypomania first-episode group, M/HF). A group of 60 healthy adult females matching general demographic data, such as race and age, were selected as the control group. Blood samples were taken from both groups to measure serum triiodothyronine (T3), thyroxine (T4), free triiodothyronine (FT3), free thyroxine (FT4), and thyroid stimulating hormone (TSH) concentrations.

**Results:**

The duration of current onset in the M/HF group was significantly less than that in the DF group (23.1 ± 20.2 vs. 125.2 ± 41.0 days). About 27% of patients in the M/HF group had thyroid abnormalities, in contrast to 60% in the DF group. The blood T3 and T4 levels in both the M/HF group and the DF group, as well as the TF3 levels in the DF group, were significantly lower as compared to control. The M/HF group had significantly higher T3 and FT3 levels than the DF group. The blood T3 levels were inversely correlated with the Young’s Mania Scale score and the Hamilton Depression Scale score in both the M/HF and DF groups.

**Conclusion:**

Thyroid dysfunction resulting in reduced levels of blood thyroid levels may be involved in the disease progression of bipolar disorder, and correlated with the clinical symptoms in patients with depression or mania as the first episode.

## Introduction

Bipolar disorder (BD) is a common psychiatric disorder with abnormally high or low affect as the main manifestation. Epidemiological data show that bipolar disorder has high prevalence (lifetime prevalence of 4.4%) and high relapse rate (90% of patients have multiple relapses) (1–3), causing a great harm to individuals, families and society. Recent studies on large samples of BD have shown a higher proportion of women in the BD population, suggesting a gradual increase in the prevalence of female BD patients (4, 5). In recent years, the relationship between thyroid gland and BD has been widely emphasized by researchers. Studies on neurotransmitters, immunology and neuroplasticity have uncovered a number of cellular and molecular mechanisms that may explain the link between thyroid dysfunction and BD. The important role of neurotransmitters (norepinephrine, 5-hydroxytryptamine, dopamine, and γ-aminobutyric acid) in the pathogenesis of mood disorders has been confirmed (6, 7). The relationship between thyroid hormones and these neurotransmitter systems not only explains the psychiatric symptoms accompanying patients with thyroid disorders, but also the thyroid dysfunction in patients with mood disorders and the therapeutic effects of thyroid hormones in patients with mood disorders (8, 9). There are some similarities in the distribution of the HPT axis and neurotransmitter systems in the brain, suggesting the possibility of interaction between the two. For example, key brain regions exist in both the HPT axis and the neurotransmitter system, and thyroid hormone receptors are widely distributed in the brain. Many limbic system structures where these receptors are located are associated with the development of mood disorders (9). Studies report that manic/light manic episodes and mixed episodes differ significantly in terms of thyroid function abnormalities (10, 11). Su Yousong et al. found that thyroid function abnormalities differed between manic/hypomanic first-episode patients and depression first-episode patients in the disease remission and episode periods, and the triiodothyronine (T3), free triiodothyronine (FT3), and free thyroxine (FT4) levels were higher in the manic/hypomanic first-episode group than in the depression-primed group during the episodic period (12). However, most of the above studies were conducted only with case groups.

The clinical presentation of bipolar disorder is complex and varied, with an average of 5 to 10 years from onset to diagnosis. Only 20% of patients with bipolar disorder are diagnosed with bipolar disorder when they seek consultation in the first year of a depressive episode (2). A study of 2,308 bipolar patients showed that the first onset of symptoms was a depressive episode in 54%, a manic episode in 22%, and a mixed episode in 24% patients (13).

The present study was designed to compare the thyroid hormone levels between cases and control groups of female bipolar disorder patients, in addition to the comparison of the thyroid hormone levels between mania/hypomania first-episode and depression first-episode subtypes. Outcomes from this study is anticipated to improve our understanding on the relationship between clinical symptoms of bipolar disorder and serum thyroid hormone levels, thereby improving the diagnosis and treatment of bipolar disorder.

## Patients and methods

### Participants

**Figure 1:**
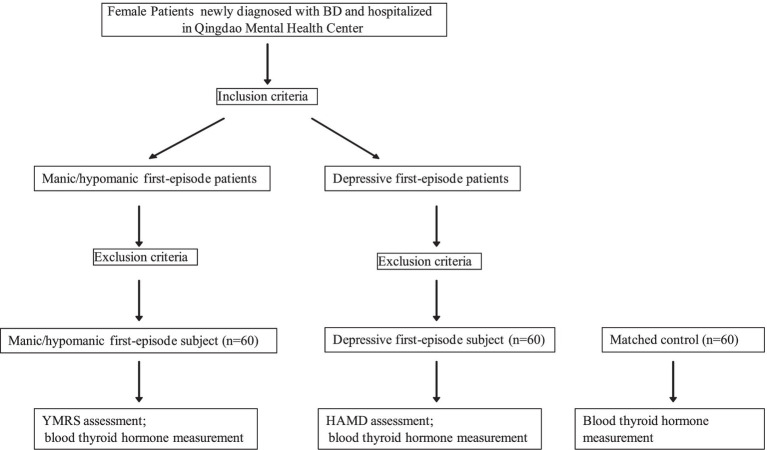
Study design. BD, bipolar disorder; RMRS, Young’s Mania Rating Scale; HAMD, Hamilton Depression Scale.

This study was approved by the Hospital Ethics Committee at Qingdao Mental Health Center (2019046). The case group was composed of 120 female patients newly diagnosed with bipolar disorder, including 60 patients of the manic/hypomanic first-episode and 60 of the depressive first-episode, recruited consecutively at Qingdao Mental Health Center from August 1, 2020 to July 31, 2021. A flow chart of the study design is shown in Figure 1. Inclusion criteria: (i) meeting the ICD-10 classification of mental and behavioral disorders: clinical description and diagnostic points of bipolar disorder manic episodes and depressive episodes; (ii) age of 18–45 years old; (iii) not receiving lithium carbonate or quetiapine that significantly affect the level of thyroid function before admission. Exclusion criteria: having (i) other psychiatric diseases; (ii) neurological diseases; (iii) serious physical diseases (12); (iv) history of drug and alcohol abuse; and (v) endocrine diseases. Disease diagnosis was made by a research physician qualified as an attending physician and reviewed by another research physician qualified as an associate chief physician.

In addition, sixty (60) healthy adult females aged 18–45 years (mean age: 30.2 ± 6.4 years) were selected as the control group. All the enrolled patients themselves or their families agreed to participate in this study and signed the informed consent form.

### Data collection

Demographic data and clinical symptoms of the enrolled patients were collected based on their medical history data and using a self-administering general condition questionnaire. All participating physicians were psychiatrists at the attending level or above and were consistently trained and familiar with the study protocol. Meanwhile, general data of the control group were collected.

### Clinical assessment of mania or depression severity

The severity of mania in the manic/hypomanic first-episode patients was assessed with the Young’s Mania Rating Scale (YMRS). The RMRS consists of 11 items and each item is scored on a 0 to 4 scale. Patients were scored by two trained raters independently, based on the on-site interview and the insiders’ information with the patients’ usual conditions as a reference. The total score of YMRS ranges from 0 to 60, with 0–5 points as normal, 6–12 points as mild, 13–19 points as moderate, 20–29 points as severe, and 30–60 points as extremely severe mania.

The 17-item Hamilton Depression Scale (HAMD) was used to assess the depressive condition in the depressive first-episode patients. Two trained raters performed the evaluation through conversation and observation, and they scored independently. Most items were scored on a 0–4 scale: 0- none, 1- mild, 2- moderate, 3- severe, and 4- extremely severe. A few items were rated on a 0–2 scale: 0- none, 1- mild to moderate, and 2- severe. The total score < 7 indicates a normal condition, 7–17 possible depression, 18–24 mild to moderate depression, and > 24 severe depression.

### Thyroid hormone level measurement

Fasting blood was taken from the patients at 7:30 am on the second day of hospitalization prior to receiving any medical treatment to test the thyroid function level, including the measurement of serum T3, FT3, thyroxine (T4), FT4, and thyroid-stimulating hormone (TSH) levels using an electrochemiluminescence method. The fasting blood of the control group was drawn within 72 h of enrollment.

### Power and statistical analysis

Our preliminary data suggested a 20–30% difference in the incidence of thyroid abnormality between the manic/hypomanic first-episode and the depressive first-episode patients. Therefore, a power analysis using an effect size of 20%, assuming a standard deviation (SD) of 30%, indicated that a group size of 60 patients would achieve a power of 0.95 to detect the difference in thyroid abnormality rate between the manic/hypomanic first-episode and the depressive first-episode groups, with a significance level of 0.05 and a two sample two tailed *t* test.

All statistical analysis was performed with the SPSS 17.0 software package. The count data were expressed as cases (*n*), and tested by using the chi-square test. The measurement data were tested for normality with both descriptive methods and Kolmogorov–Smirnov and Anderson-Darling Tests. Then the data were expressed as mean ± SD, and tested by one-way ANOVA for multiple comparisons. If a significant difference existed, Levene’s test was used to test the consistency of variance, followed by the Least-significant difference (LSD) method for paired test if the variance was consistent, or Tamhane’s T2 test if the variance was inconsistent. The comparison between two means was performed by independent samples *t*-test. *p* < 0.05 indicates that the difference is statistically significant, and *p* < 0.01 indicates that the difference is highly statistically significant.

## Results

### Comparison of general demographic and clinical information

A total of 120 patients with bipolar disorder were enrolled as the case group, and there was no significant difference in the age of disease onset between the manic/hypomanic first-episode group and the depressive first-episode group (Table 1). There was no significant difference in height or weight between these two groups. However, the duration of this episode (days) in the manic/hypomanic first-episode group was significantly shorter than that in the depressive first-episode group (Table 1).

**Table 1:** Demographic and clinical characteristics between the manic/hypomanic first-episode group and the depressive first-episode group.

	Manic group (mean ± SD)	Depression group (mean ± SD)	*p*
Age (years)	31.3 ± 9.4	29.9 ± 12.8	0.609
Height (cm)	164.2 ± 9.2	164.5 ± 10.1	0.976
Body weight (kg)	62.3 ± 13.1	61.4 ± 14.2	0.560
BMI (kg/m^2^)	22.3 ± 3.3	21.4 ± 4.2	0.519
Duration of current episode (days)	23.1 ± 20.2	125.2 ± 41.0	0.003
Young’s Mania Scale score	21.0 ± 3.2	__	__
Hamilton Depression Scale score	__	28.0 ± 4.9	__

### Comparison of thyroid hormone levels

Thyroid abnormalities/dysfunctions were present in approximately 27% of the manic/hypomanic primed group and in approximately 60% of the depressed primed group. The serum levels of T3 and T4 levels were significantly lower in the manic/hypomanic first-episode group than that in the normal control group. Likewise, the levels of T3, T4, as well as the FT3 levels, were significantly lower in the depression first-episode group than the control. As compared to the depressive first-episode group, the manic/hypomanic first-episode group had significantly higher serum levels of T3 and FT3, while the levels of T4 and FT4 were comparable between these two groups (Table 2).

**Table 2:** Thyroid hormone levels in the two subgroups of patients with bipolar disorder and in the normal control group (mean ± SD).

Group	Number of participants	T3 (nmol/L)	FT3 (pmol/L)	T4 (nmol/L)	FT4 (pmol/L)	TSH (μIU/ml)
Manic / hypomanic first-episode group	60	1.77 ± 0.44^a^	4.69 ± 1.32^a^	99.94 ± 27.51^a^	17.16 ± 5.62^a^	3.05 ± 3.38^a^
Depression first-episode group	60	1.57 ± 0.37^b^	4.14 ± 0.74^b^	97.51 ± 28.26^a^	16.50 ± 3.42^a^	2.24 ± 0.32^a^
Control group	60	2.20 ± 0.01^c^	4.95 ± 0.42^a^	123.5 ± 5.32^b^	17 ± 0.32^a^	2.24 ± 0.42^a^
*F*		31.293	7.292	13.118	0.267	1.002
*p*		0.000	0.001	0.000	0.766	0.371

Different letters in each hormone category indicate significant difference between groups.

Consistently, when stratifying the serum thyroid hormone levels, a significantly higher frequency of low T3 and T4 levels was observed in both the manic/hypomanic first-episode and the depression first-episode groups, and a significantly higher frequency of low FT3 in the depression first-episode group, as compared to control (Table 3). In addition, the depression first-episode group had significantly higher frequency of low T3 and FT3 levels than the manic/hypomanic first-episode group (Table 3).

**Table 3:** Incidence of thyroid dysfunction in the manic/hypomanic first-episode group and the depressive first-episode group.

	Manic group (*N*)	Depression group (*N*)	Control group (*N*)	*p*
**T3** (1.3–3.1 nmol/L)^*^:				
< 1.9 nmol/L	38	48	25	0.035
1.9–2.5 nmol/L	22	12	35	
> 2.5 nmol/L	0	0	0	
**FT3** (3.1–6.8 pmol/L)^*^:				
< 4.3 pmol/L	20	30	14	0.039
4.3–5.6 pmol/L	32	28	45	
> 5.6 pmol/L	8	2	1	
**T4** (66–181 nmol/L)^*^:				
< 104 nmol/L	34	32	26	0.043
104–143 nmol/L	22	24	32	
> 143 nmol/L	4	4	2	
**FT4** (12–22 pmol/L)^*^:				
< 15 pmol/L	24	16	9	0.078
15–19 pmol/L	22	30	47	
> 19 pmol/L	14	14	4	
**TSH** (0.27–4.2 μIU/ml)^*^:				
< 1.6 μIU/ml	22	24	11	0.384
1.6–2.9 μIU/ml	22	16	38	
> 2.9 μIU/ml	16	20	11	

^*^Normal reference range.

### Correlation between thyroid hormone levels and disease severity

We performed a correlation analysis between the five thyroid hormone levels in the mania group and the Young’s mania scale score, and found that the lower the T3 level, the higher the Young’s mania scale score which suggests a more severe condition, with a correlation coefficient *p* = −0.354. Similarly, an inverse correlation was also observed between the T3 levels in the depression group and the Hamilton depression scale score, that is, the lower the T3 level, the higher the Hamilton depression scale score which suggests a more severe disease, with a correlation coefficient *p* = −0.479.

## Discussion

Abnormalities in the hypothalamic–pituitary-thyroxine (HPT) axis are common in bipolar disorder (14), and some studies have shown that the thyroid hormone levels are 2.55 times more likely to be abnormal in patients with bipolar disorder than in the healthy population (15). In the present study, we found that thyroid abnormalities/dysfunction were present in approximately 27% of the manic/hypomanic first-episode group and in approximately 60% of the depression first-episode group, which supports the previous findings and suggests that thyroid problems may play an important role in the disease progression of bipolar disorder.

Our study found that the episode duration in the bipolar disorder patients with depression as the first onset was much longer than that in patients with mania/hypomania as the first onset. We also found that the T3 and FT3 levels in bipolar disorder patients with depression as the first onset were significantly lower than that of mania/hypomania first onset patients. These results suggest that the HPT axis may be more severely abnormal in depression first-episode bipolar disorder patients. As a result, these patients are more prone to anxiety, depression and mood instability, thus maintain the depressive episodes longer.

In contrast to the T3 and T4 levels which were significantly lower in bipolar disorder patients with either mania/hypomania or depression as the first onset compared to control, a significantly lower level of FT3 was only observed in patients with depression as the first onset, which suggests that there may be different biological mechanisms responsible for the different first onset subtypes of bipolar disorder.

We also found that there is a correlation between thyroid hormone levels and clinical symptoms of bipolar disorder, that is, the lower the T3 levels are, the more severe the symptoms of bipolar disorder tend to be, in both the mania/hypomania and depression first groups. Therefore, in clinical practice we may need to take into account the thyroid hormone levels in bipolar disorder patients when choosing therapeutic drugs (16). A precaution needs to be taken since some clinical drugs may cause thyroid abnormalities. For example, the commonly used mood stabilizer lithium carbonate can inhibit thyroid hormones by inhibiting the coupling of iodinated tyrosine, inhibiting iodine uptake, altering the structure of thyroglobulin, reduce thyroxine release, interfering with T3 and eT4 deiodination, altering cellular response to thyroid response of the thyroid, etc. A previous study showed that 0–47% (mean 10%) of patients on long-term lithium carbonate had hypothyroidism (14).

Several studies have shown encouraging results by targeting thyroid hormones in treatment of BD. In the Canadian Mood and Anxiety Treatment Network 2018, it is mentioned that thyroid hormones can be used as a third-line combination therapy drug for the acute treatment of biphasic type II depression (17). Clinically, it is often found that many BD patients with thyroid hormone levels close to normal low values have difficulty in achieving optimal treatment outcomes. It has been found that T3 not only enhances the therapeutic effect of antidepressants in refractory bipolar depression, but also has a potentiating effect on lithium salts, and that it has a better therapeutic effect in rapid-cycling BD (18). The use of supraphysiologic doses of T4 as an adjunctive therapy in patients with refractory BD depressive episodes with normal thyroid function shortened the duration of depression (19), and T4 as an adjunctive therapy was also effective in the maintenance treatment of patients with major depressive episodes or schizoaffective disorder and in the maintenance treatment of refractory rapid-cycling and non-rapid-cycling BD (20, 21). The use of supraphysiologic doses of L-T4 appears not to increase anxiety symptoms, suggesting that supraphysiologic doses of L-T4 may be a choice for patients with severe anxiety symptoms at baseline, and for the use of patients with refractory bipolar depression and co-morbid anxiety symptoms (22). Other studies have also shown that the use of exogenous thyroid hormones in BD treatment did not lead to the development of hyperthyroid complications, although it led to higher levels of thyroid hormones in the body (23). These studies together with our findings provide valuable information for future clinical practice to improve the treatment of BD particularly in patients with thyroid abnormalities.

## Limitations

There are several limitations with this study. Firstly, the sample size of this study is relatively small, which limited us from further data analysis with stratification factors such as age. Age may be a confounding factor in the analysis of thyroid hormone levels in bipolar patients. However, our present data indicated that the age distribution was not significantly different between the manic/hypomic first-episode and depressive first-episode groups, which may minimize the influence of age on the results. Secondly, patients were from the same psychiatric hospital, so regional bias is possible. Thirdly, this study only included the manic/hypomanic episode and depressive episode subtypes of BD, without considering the mixed episode, which may bias the study results. Future work is needed with larger sample sizes and including the mixed episode subtype, to confirm results from this study.

## Conclusion

In conclusion, results from this study demonstrate a decrease in certain types of thyroid hormones in patients of bipolar disorder compared to normal control. The patterns/levels of thyroid hormones involved were different in two subtypes of bipolar disorder with a correlation between thyroid hormone levels and clinical symptoms of the disease. We anticipate this information will assist in the improvement of clinical practice in treatment of bipolar disorder patients with thyroid abnormalities.

## Data availability statement

The original contributions presented in the study are included in the article/supplementary material, further inquiries can be directed to the corresponding author.

## Ethics statement

Written informed consent was obtained from the individual(s) for the publication of any potentially identifiable images or data included in this article.

## Author contributions

XS and YF designed the study, collected, and analyzed the data. XS and LY wrote the first draft of the manuscript. YL and BZ discussed and commented on the manuscript. All authors contributed to the article and approved the submitted version.
